# Supplementary figures and images for: Long-term genomic surveillance reveals the circulation of clinically significant Salmonella in lymph nodes and beef trimmings from slaughter cattle from a Mexican feedlot
Source: PLoS One. 2024 Oct 18;19(10):e0312275. doi: 10.1371/journal.pone.0312275 (PMC11488740; doi:10.1371/journal.pone.0312275)

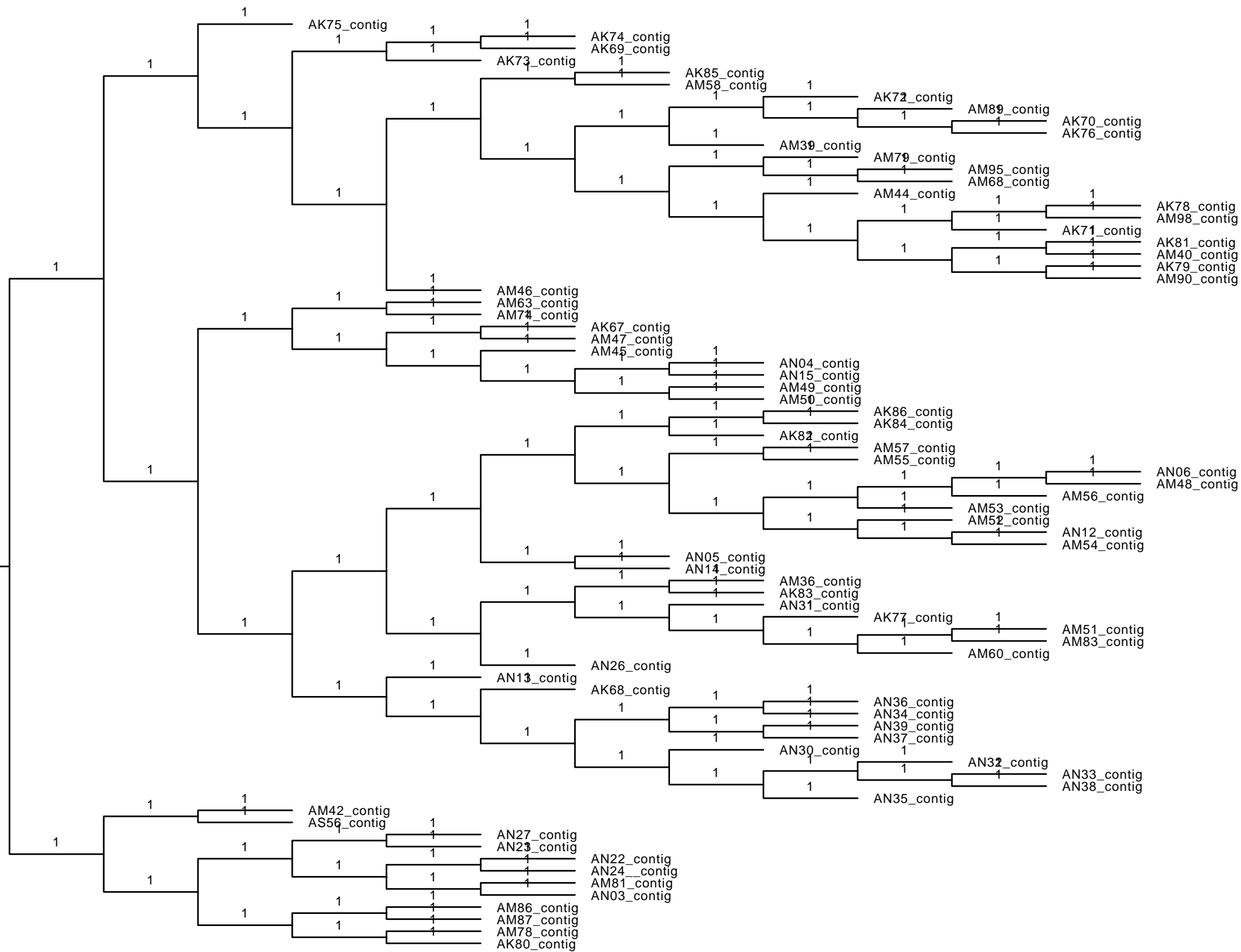

2.0

Supplement: S2 File — (PDF) [file pone.0312275.s002.pdf]
