# Supplementary material for: Long-term genomic surveillance reveals the circulation of clinically significant Salmonella in lymph nodes and beef trimmings from slaughter cattle from a Mexican feedlot
Source: PLoS One. 2024 Oct 18;19(10):e0312275. doi: 10.1371/journal.pone.0312275 (PMC11488740; doi:10.1371/journal.pone.0312275)
Supplement: Attachment — Submitted filename: Response to reviewers R1.docx [file pone.0312275.s005.docx]

PONE-D-24-16621

Long-term genomic surveillance reveals the circulation of clinically significant *Salmonella* in lymph nodes and ground beef from healthy cattle from a Mexican feedlot (updated title)

PLOS ONE

The authors thank the reviewers for their thorough revision and valuable comments that helped improve our manuscript. Below we list each comment raised during the reviewing process, followed by the authors’ responses**.**

**Responses to editor’s comments (EC)**

**EC1.** *Please ensure that your manuscript meets PLOS ONE's style requirements, including those for file naming. The PLOS ONE style templates can be found at*

https://journals.plos.org/plosone/s/file?id=wjVg/PLOSOne_formatting_sample_main_body.pdf and

https://journals.plos.org/plosone/s/file?id=ba62/PLOSOne_formatting_sample_title_authors_affiliations.pdf

**Authors’ Response (AR)1.** We carefully reviewed the style requirements to make sure the manuscript meets them.

**EC2**. *Please include captions for your Supporting Information files at the end of your manuscript, and update any in-text citations to match accordingly. Please see our Supporting Information guidelines for more information:* <http://journals.plos.org/plosone/s/supporting-information>

**AR2.** We made these corrections. Please, refer to the revised manuscript.

**Responses to reviewers’ comments (RC)**

**Reviewer # 1**

***RC1****. In this study, Delgado-Suárez et al. compared the* Salmonella *isolated from lymph nodes and ground beef to address the potential clonal distribution of* Salmonella *found in animals and food products. Overall, the idea is valuable as it aims to address the potential public health risk associated with cattle lymph node carriage and ground beef consumption. However, the methodology and approach do not fully answer the research question. Please see my specific comments below.*

**AR1**. We appreciate these comments and the thorough revision of our manuscript.

***RC2****. Lines 22-23: The phrase “we used” is vague and almost sounds like they “spiked” these bacteria. They further analyzed the data obtained from these strains. They need to clarify this.*

**AR2.** The authors agreed to adjust the text to avoid confusing readers. Thus, the whole abstract was modified.

***RC3.*** *The overall introduction needs improvement. It is unclear if these serotypes are public health threats, the reasoning behind the selection of the strains, and if they cause clinical symptoms in cattle or disease in humans. Several papers have explored the clonal distribution of Salmonella in cattle lymph nodes, other samples, and the environment. Compared to those, what is the difference in this study? What are the specific aims? What is the research gap in the field? Are there any similar studies? Additionally, it is unclear how these isolates were obtained.*

**AR3.** We should have stated that all the serovars mentioned in the introduction have been linked to human infections in Mexico and other countries. We did not do it since the introduction starts highlighting the implication of ground beef in human salmonellosis outbreaks (L41-42). This is now mentioned in the updated version of the manuscript.

Moreover, we did not select any strain for this research. We guess we confused this reviewer by using the phrase “set of *Salmonella* isolates” in L62. However, we used all the strains that we collected during the survey previously conducted by our research group (cited in L63). The text was corrected to gain in clarity.

In the paragraph of L50-60, we mentioned previous research in the topic and commented the research gaps (L51 onwards). This section was modified to better address these topics and provide a clearer justification of the research. Please, refer to the revised manuscript.

We will not provide here (in the introduction) the full explanation of how the isolates were obtain since that is part of the methods. However, we adjusted the text to ensure the introduction fulfills its purpose.

***RC4****. Lines 69-73: The authors should clarify why these 77 isolates were selected and what their source distribution is. What is the purpose of adding one isolate from one serotype when the overall goal was to compare the strains obtained from lymph nodes and ground beef? What type of lymph node is it, and how were they collected? What is the relationship between the ground beef and the lymph node? Were they obtained from the same animals? The carcass retailer in Mexico City was the source of both lymph nodes and the ground beef. What is the location of the study? How were these samples selected? Were they from the same season or batch? Important study design-related information needs to be provided for clarity. Also, how were these strains isolated? Were the same isolation methods used? How were they stored?*

**AR4**. We agree these details are absolutely required. We did not describe them in full to avoid repeating ourselves with the description provided in previous papers of the same project that we have published (i. e. citation #9 in L74). However, we agreed to describe all the methods here as well, for the sake of clarity. Please, refer to the revised manuscript.

***RC5****. Lines 79-80: The methods provided are unclear. How many colonies? What is the volume of TSB? How long was the incubation? Additionally, the authors should provide the brand, city, and country information for the materials used in the study. Was the DNA quality assessed? If so, how? Which kit was used for Qubit? The entire materials and methods section should be revised to ensure proper detailed information is provided for the repeatability of the work performed. How was the serotyping performed?*

**AR5**. To ensure clarity and reproducibility, this whole section (L79-95) was adjusted. Please, refer to the revised manuscript.

***RC6.*** *Lines 85-87: What is the Nextera XT version 3 kit? Both library and sequencing kit information should be provided.*

**AR6**. The Illumina Nextera XT version 3 kit is a genomic DNA library preparation kit, which is normally cited this way: the Illumina kit being used and the sequencing conditions that complement that information (L86-87: paired end, 2 x 150 bp insert size). We have published papers with this description in other journals (doi:10.1038/s41598-018-28169-4, doi: 10.1128/aem.02149-21) and Plos One as well (doi: 10.1371/journal.pone.0243681). There are many examples from other authors that use the same standardized description. Some authors describe details of DNA library preparation but only when they made modifications to the Illumina protocol, which is publicly available for downloading (<https://genome.med.harvard.edu/documents/libraryPrep/IlluminaNexteraXTProtocol.pdf>). We did miss mentioning it was an Illumina kit, and that we followed manufacturer’s instructions, though. In any case, we corrected this whole section in response to ***RC6***.

***RC7.*** *Line 87: It is unclear why 30x coverage was aimed for. Please provide the reasoning with a citation.*

**AR7**. Previous research has demonstrated that *Salmonella* draft genomes with a sequencing depth of coverage of 15-20x can be accurately placed in a phylogenetic context (doi: 10.1186/1471-2164-13-32). However, our laboratory is a contributor of the FDA’s Genome Trakr program, which has the 30x cutoff value established in a published protocol (<https://doi.org/10.1007/978-1-4939-9000-9_22>, see Table 2). We did not cite any of these sources. Hence, the corrected version includes now a citation to the Genome Trakr protocol.

***RC8.*** *Line 93: What are the criteria?*

**AR8**. The text was adjusted to describe in full the trimming strategy we followed.

***RC9.*** *Line 100: Please provide brief information rather than referring to previous papers for methodologies important for this paper.*

**AR9**. This information is not actually methodology. These are data of genome assembly quality attributes. They can be consulted by interested readers but are not necessary to replicate the research. We cited our previous paper since these data correspond to the same genomes used in this study, were published as supplementary information, and had a DOI assigned (<https://doi.org/10.1371/journal.pone.0243681.s001>). Considering this reviewer comment, we provided a summary of genome assembly quality attributes within the manuscript (Table 1) and cited the DOI of the full dataset.

***RC10.*** *Line 101: Why was the genome annotation tool used? It is unclear. If the purpose is to look for virulence genes, why not use a well-known, regularly maintained virulence gene database?*

**AR10**. We did use databases: the virulence factors database (for virulence genes, as stated in L121) and the pathogenicity islands database (for pathogenicity islands, as stated in L131). Moreover, for the purpose of our research, we prefer working with the annotated genomes. In our experience, this method yields more reliable results, as explained below, and thus we prefer to keep it as it is.

For instance, when predicting the presence of pathogenicity islands using SPIFinder, you get a highly fragmented and incomplete output. The screenshot below shows an example output fragment of SPIFinder for one of our genomes (NCBI accession [GCA_007738755.1](https://www.ncbi.nlm.nih.gov/assembly/GCA_007738755.1)). The tool identified seven small fragments of SPI-1 ranging from 259 to 3,155 bp). However, the total size of SPI-1 is >44 Kb (from *fhlA* to *mutS*).

Even if using the SPIFinder database on the command line, thresholds need to be specified for identity percentage and coverage match. Hence, having a positive hit does not tell the whole story, unless the whole SPI is represented in the genome, which is not often the case as variability in SPIs is common across *Salmonella* subpopulations.

For this reason, we prefer to use a BLAST atlas analysis, which portraits a comparison of the full reference SPI sequences in a group of genomes. This approach has been used for over a decade (see 10.1007/s00248-011-9880-1) and allows more meaningful comparisons. This is important considering SPIs are horizontally acquired and several of them are unstable in certain regions. Moreover, we can check the accuracy of the prediction by browsing through the genome features and verifying that their genomic context match that of the reference sequence.

For individual virulence genes, we can also conduct BLAST-p search analyses within the annotation server using a very low e-value threshold (e.g., 10^-30^), whereby matching sequences are mapped back to the corresponding genes in The Seed Viewer. In case of ambiguous annotations or amino acid identities below 90%, it is possible to perform a Psi-BLAST analysis, within The Seed Viewer, to confirm whether the matching protein was a homolog of the reference protein.

Despite we decided to keep the methods we used unchanged, we adjusted the text to ensure these procedures are fully understandable and reproducible. Moreover, we repeated the BLAST atlas analyses in the newly created proksee server and generated new figures. We had to do this because the former GView web server was shut down. We are now reporting the profile of the 12 SPIs in Fig 3 and included three additional figures (Fig 4-6) to provide a more detail analyses of the variations observed within individual SPIs.

***RC11.*** *Line 103: How do the authors use the terms "phylogenetic" and "evolutionary analysis"? Are they the same? Can the authors discuss the evolutionary direction and SNP/time differences observed?*

**AR11**. While phylogenetic analysis represents the evolutionary relationships among organisms, the term “evolutionary analysis” is broader in nature, as it may address several areas of interest. In our study, we were interested in assessing if there was a linkage among the ecological niche (i. e. isolation source) and the evolutionary relationships depicted in the phylogenetic tree. That is why we used the Mesquite program, which allows integrating genetic information with other types of data: phenotypic, location, isolation source (the metadata of our interest that was defined as the “character state” for the analysis with Mesquite, L115-116), among others.

To avoid confusing readers, the two analyses are described under different subheadings in the updated manuscript. We also decided to use a new term “reconstruction of ancestral isolation sources” to be more specific and gain in clarity. This comment refers to the methods section. However, we agreed to include and discuss the SNP differences observed in the results and discussions sections, respectively.

***RC12.*** *Line 105: Why did the authors prefer using assembled genomes?*

**AR12**. We conducted a SNP-based phylogeny using whole genomes, which is a computationally demanding task. For that purpose, we used the highly cited CSI Phylogeny methodology (see <https://doi.org/10.1371/journal.pone.0104984>) as in previous papers that we have published. This program may use either raw reads or assembled genomes. We preferred to use the assemblies to avoid the drawbacks associated with raw reads: their huge file size and the long time required for task completion. We are aware that there are other programs that perform the analysis using the raw reads as input (e.g., CFSAN SNP pipeline, and others). But then again, it is more difficult for us to use these programs due to the bulky size of the reads and the time require to upload them to the servers that provide free access.

***RC13.*** *Lines 105-107: It is very unclear how the trees were generated, how the alignments were made, which matrices and models were used for tree inference, and how they were selected.*

**AR13***.* As declared in the methods section (L110), we only used the CSI Phylogeny program to obtain the multiple genome alignment. This part of the analysis is described in the CSI Phylogeny paper that we cited (<https://doi.org/10.1371/journal.pone.0104984>). We did not make any modifications to the code as we used the program online. Although CSI Phylogeny also infers trees using FastTree, RAxML performs better than FastTree without the need of modifying its code (as recognized by the same authors of CSI Phylogeny, see <https://darlinglab.org/blog/2015/03/23/not-so-fast-fasttree.html>). Therefore, after obtaining the alignment, we uploaded it to the CIPRES Science Gateway server and analyzed it using the maximum likelihood method (RAxML program) under the GTR model of nucleotide evolution using default values. To provide a more precise description of the methods, we are now describing the default values of each program, and their URLs. We believe these changes fulfill the purpose of allowing reproducibility.

***RC14.*** *Line 107: What is the reason for using a Typhi strain as a reference? This is interesting. Please provide reasoning.*

**AR14.** The program we used to align the genomes (CSI Phylogeny) uses a reference-based approach (only one reference genome can be indicated). However, it does not force query genomes to match the sequence of the reference genome. It only happens when using raw reads as input, which was not our case. We used *de novo* assembled genomes, whereby the program (CSI Phylogeny) calculates the SNP distance between each query genome and the reference genome. This allows calculating the SNP distance between any two genomes (all possible pairs) and building the SNP matrix, which is one of the output files. Hence, we could have selected any genome as a reference as it will not change the results. However, we chose that of *Salmonella* Typhi to use it as an outgroup in the phylogenetic analysis. The SNP matrix, which will be published as supplementary information if this paper is accepted, reports a SNP distance between the study genomes and that of Typhi of 43,414–53,121 SNPs, which is consistent with the genetic distance between typhoidal and non-typhoidal strains.

In any case, we double checked this issue by running intraserovar analyses using as a reference a closed genome of the same serovar, as well as using the type strain Typhimurium LT2. The results were practically the same. For instance, in our original phylogenetic analysis, using the Typhi CT18 strain as a reference, the Typhimurium isolates were divided into two clades: one with a singleton (AK68 strain name), which was nearly 500 SNPs from the remaining nine isolates that clustered together and were within 0-24 SNPs from each other, as shown in this fragment of Fig 1:

In the repeated intraserovar analysis using the LT2 strain as a reference (instead of Typhi), apart from the improved resolution that can be expected, the results were the same. Again, the AK68 was separated from the other nine Typhimurium isolates (613-628 SNPs), which were also very close to each other (0-25 SNPs):

The same occurred with isolates of other serovars. To avoid confusing readers, the updated manuscript now declares that the Typhi strain was used to align the genomes and as an outgroup for the phylogenetic analysis. Moreover, the updated phylogenetic tree includes the Typhi strain, although no virulence data for this strain is reported in Fig.1.

***RC15.*** *Line 111: Why were these models selected? What are the default values?*

**AR15.** The GTR +Γ model is a standard model used for maximum likelihood inferences (see <https://doi.org/10.1186/s12859-016-0985-x>). In the just cited paper, the authors conducted a comprehensive analysis using 39 empirical datasets. It showed that, in most cases (95%), there is no difference between trees inferred under the standard GTR +Γ model and trees inferred under the best-fit model selected among all possible time-reversible DNA substitution models based on the AIC (Akaike Information Criterion), AICc (corrected AIC) BIC (Bayesian Information Criterion), and DT (Decision Theory) criteria. Therefore, we believe using the standard model was a good choice for this research, both considering the reasons mentioned above and the fact that *Salmonella* is a clonal organism. As mentioned in **AR14**, the default values were included in the updated manuscript.

***RC16.*** *Lines 116-117: Please clarify your reasoning for using the parsimony unordered model. What is the goal here, and how were the parameters selected? What parameters were used? How was this tree constructed?*

**AR16***.* The Mesquite program is not designed to infer phylogenetic trees, but rather for diverse analyses, such as character state evolution, using already inferred trees. The term “character state” refers to metadata of many sorts (measurements, observations) that can be attached to taxa. This way, the program uses the inferred phylogeny to estimate the most likely “state” of the ancestors. In other words, it infers the history of character state evolution.

In our research, we had a phylogenetic tree of 77 isolates originated from two different sources: lymph nodes and ground beef. The isolation source was the character state of our interest, and this information was attached as metadata to the phylogenetic tree. We used the parsimony unordered model since it is the one that should be used for categorical characters such as the isolation source (see Mesquite’s documentation webpage (Analyses/Character evolution link):

<https://www.mesquiteproject.org/Character%20Evolution.html?TaxaTreesCharactersPanel=open&AnalysesPanel=open>

In summary, having a phylogenetic tree and a distribution of character states in the observed (terminal) taxa, Mesquite reconstructs the character states at ancestral nodes. It also paints the tree to show ancestral states and includes a trace legend within the output.

Through this analysis, we were able to estimate the isolation source of the ancestors of our study isolates across the tree. The goal was to assess to which extent the isolates found in ground beef samples had evolved from isolates collected from lymph nodes or vice versa. This information cannot be derived from the phylogenetic analysis alone. It only provides the hypothesis that isolates from both sources are close to each other, but it is not possible to determine what is the directionality.

We adjusted the methods section to provide more details of this analysis. We are now including the phylogenetic tree (as a supplementary file) and the Mesquite documentation URL to allow for a full reproducibility of this analysis (refer to the revised manuscript).

***RC17.*** *Line 124: Protein or gene? Also, why not use VirulenceFinder? How was the threshold value determined? How did the authors ensure the database used was up to date with the correct sequence information, including the specific variants of the genes? What does “resulting amino acid percentage” mean? Please clarify.*

**AR17**. We used the set of genes of the Virulence Factor Database (VFDB) but the BLAST analysis was performed with their amino acid sequences (BLAST-P), which are also available for downloading at the VFDB website. The text was adjusted to make this clear.

The VirulenceFinder does not include data for *Salmonella*. Currently, it can only be used for *E. coli*, *Enterococcus*, *Listeria* and *S. aureus* (see <https://cge.food.dtu.dk/services/VirulenceFinder/instructions.php>).

The VFDB has been active since 2005. It is used by important global companies such as Qiagen, and it is widely used in scientific publications worldwide (2,889 citations of the papers reporting database updates during 2016-2022).

This research was conducted during 2017-2018. At that moment, we used the newest database version (2016). However, the COVID-19 pandemic delayed the drafting of this manuscript. Thus, we repeated the analysis with the 2022 version, but forgot to cite it. This citation was updated in the adjusted manuscript.

Within RAST, even when choosing the default e-value threshold (10), the output includes the bit score and e-value of each positive hit, but it produces many hits that make no sense. As shown in the screenshot below, you can get as much as 10 positive hits when using the default (10) e-value threshold (this is an example run for the amino acid sequence of the *fur* gene):

To avoid wasting time filtering random hits, we chose a very stringent e-value threshold (10^-30^). This information was included in the updated manuscript.

To build the virulence profile of our annotated genomes, we conducted a BLAST protein analysis using as a reference the amino acid sequence of *Salmonella* major virulence factors that are available from the VFDB. The output of this analysis provides the amino acid identity percentage between the reference protein and its positive hits in the genome (see below):

The text was adjusted to ensure it is clear what we mean with “the resulting amino acid identity percentage”.

***RC18.*** *Line 130: I suggest the authors use an already compiled, known database rather than manually collecting the reads, as it will lack validation. If manually curated data will be used, please provide the database used as supplemental material so your work can be repeated by others. I would still suggest cross-checking findings with an online, well-known tool such as SPIFinder.*

**AR18**. We did not collect the reads. We collected the complete SPIs reference sequence from the Pathogenicity Island Database (PAIDB), which is also an online, well-known tool (<https://doi.org/10.1093/nar/gku985>). Its paper has been cited more than twice (119 times) compared with that of SPIFinder (53). Moreover, we explained before (see **AR10**) what are the drawbacks of using SPIFinder considering our study objectives and why using PAIDB provides more reliable results. In the corrected version, we are now providing the accessions of the SPIs sequences, to ensure reproducibility.

***RC19.*** *Line 131: Please explain the BLAST atlas analysis and criteria used for the search.*

**AR19**. This analysis is now fully explained in the corrected manuscript.

***RC20***. *Line 135: What are the SNP clusters submitted, and how were they analyzed on this webpage? Please provide details.*

**AR20**. The NCBI Pathogen Detection website uses the genomes that are uploaded to the database to generate a phylogenetic tree. Since the number of genomes steadily accumulates (there are currently > 600,000 *Salmonella* genomes in the database), this phylogenetic tree is so huge that it cannot be visualized in full. Therefore, each uploaded genome receives a SNP cluster accession number (if it falls within any of the SNP clusters within the tree). Upon clicking this accession number, the platform shows the fragment of the tree containing all the isolates that clustered together with that isolate. In this way, the web site facilitates researchers getting meaningful and useful information from the tree.

We did not provide this explanation considering that readers interested in comparative genomics should be familiar with the NCBI pathogen detection website. However, in the original manuscript, we provided the full list of the SNP clusters accessions as supplementary information (L138-139, S1 Table). Thus, any interested person can consult each SNP cluster, including those depicted in Figure 4. Although this comment refers to methods, perhaps the poor quality of the figures in the built PDF did not allow reviewers to fully understand this analysis. It is important to note that all we did was to consult the NCBI phylogenetic tree to check whether our isolates were close to clinical strains (i. e. if they share the same SNP cluster and what was the SNP distance to clinical strains from around the world). We adjusted the text of this section to provide a clearer explanation of this analysis.

***RC21***. *Line 143: What does “relatively diverse” mean?*

**AR21**. The phylogeny divided the isolates into two clades only (which could be interpreted as low diversity, genetically speaking). However, both clades were further split into several subclades, with many of them having splitting events, which is an indication of genetic variability. That is why we used the term “relatively diverse”. However, we agree this term is ambiguous. Hence, the text was adjusted to ensure only factual and objective information is reported. Following a previous suggestion made by this reviewer (***RC11***), the results section now includes data on the SNP distance between the study isolates.

***RC22*.** *Lines 144-150: If the authors are looking into clonal distribution, they need to start with core genome MLST, not the serotypes. We expect serotypes to cluster together. This is known knowledge. Authors should explore 7-gene MLST AND cgMLST when they explore clonal distributions*.

**AR22**. We disagree with this observation. We did not start with serovars. We are just describing the results of the phylogenetic analysis, which shows that isolates from the same serovar clustered together, except for those of Give and Muenster that clustered together despite being different serovars.

Secondly, we conducted the SNP phylogeny with whole genomes, which provides an extreme discriminatory power (compared with any other current method, including 7-gene MLST and cgMLST). In fact, we already conducted a 7-gene MLST analysis with these same genomes, (it is reported in one of our previous papers <https://doi.org/10.22319/rmcp.v11i3.5516>), and all isolates of the same serovar satisfied the clonality criteria (shared at least 6 out of the 7 alleles). Conversely, our SNP phylogeny showed that not all isolates of the same serovar are clonal. The criteria that have been set to consider clonality between isolates based on SNP phylogeny with whole genomes are far more rigorous (<https://doi.org/10.3389/fmicb.2018.01482>):

- SNP distance: ≤20
- Bootstrap support: ≥90
- Tree topology: monophyletic

Moreover, we were not looking just for clonal distributions. We also wanted to assess the evolutionary relationships among isolates from lymph nodes and ground beef that were collected and analyzed separately. In addition, we needed this specific tree as it is required as input for the reconstruction of character state (isolation source) evolution analysis (see **AR16** above).

***RC23*.** *Line 168: This paragraph reads like a discussion, not results.*

**AR23**. We mostly disagree with this observation. This paragraph describes the virulence genes that were present or absent in the study genomes, as depicted in Figure 1. Therefore, these are results of the heatmap, not discussion. The actual discussion of virulence gene profile results is between L260-276 of the original manuscript. In the paragraph quoted by the reviewer, we are not analyzing or discussing the differences observed in the virulence gene profile. We believe the last sentence (L176-179) is in the borderline of results and discussion. It reports the detection of the *st313-td* gene in the genome of one isolate of serovar Muenster (this is a result). We then mentioned that this gene has been observed to confer invasive phenotypes in strains of serovars Typhimurium and Dublin (this reads like discussion, but we thought it was convenient to inform the readers what is the role of this virulence factor that we found in the genome). Following this reviewer’s observation, we decided to remove the last part of this sentence (L177-179) from here and relocate it to the discussion section.

***RC24****. Line 180: Of course, this is a known trait of serotypes.*

**AR24**. We partly agree with this observation. However, it will remain as it is for two reasons. Firstly, there is evidence of intraserovar divergence (10.1371/journal.pone.0055687, 10.1371/journal.pone.0161225, 10.1038/s41598-018-28169-4, 10.1128/aem.02149-21). Hence, we think it is important to state that the results were uniform within serovars, and that they are reported with a representative isolate of each serovar and not for the 77 isolates, which would also make the figure unnecessarily big in size. Secondly, discussing the results should be avoided, as requested by this reviewer in the previous comment (**RC23**).

***RC25***. *Tables and graphs: The images are of poor quality and hard to read.*

**AR25.** We are sorry about that, and we understand this poor quality led to several reviewers’ remarks. Our original images are of good quality. After running them through the PACE tool, we got TIF images of good quality as well. However, the ones included in the built PDF got blurred during the rendering process. We consulted the journal office, and they told us this is the way the PDF building works. However, reviewers and editors can always download the original figure from the blue download link on top of each figure and visualize it in its full resolution. We made many tests with the figures before submitting the updated manuscript but to no avail. No matter how good the original resolution is, they always get blurred in the built PDF. Please, do not forget to download the figures this time.

**Reviewer # 2**

***RC26****.* *The objectives of this study were to compare the genomes of* Salmonella *from ground beef and lymph nodes. Title should include Mexico to give geographical context.*

**AR26.** We accepted this suggestion. This is the update title:

“Long-term genomic surveillance reveals the circulation of clinically significant *Salmonella* in lymph nodes and ground beef from healthy cattle from a Mexican feedlot”

***RC27****. Introduction. Overall the introduction is very broad and needs to include more specific examples from the literature. You include citations but do not go into detail on how any of those citations support your broad statements.*

**AR27.** This remark agrees with those made by reviewer #1 for the introduction, which was adjusted to ensure it fulfills its purpose: stating the importance of the topic, explaining the research gap being addressed (justification of the research), and stating the study objectives.

***RC28****.* *Lines 43-45. What do you mean by several Salmonella strains use certain niches in cattle. Are these particular outbreak strains or serovars. This is a broad/vague statement. Can you provide a specific example from one of the citations?*

**AR28.** This comment is related to the previous one. Please, refer to the updated introduction to check the corrections we made.

***RC29****. Lines 53-55. Is there research that particularly shows that these strains predominate in the LNs or are these strains just generally more prominent in cattle in general?*

**AR30.** Yes. We cited the research showing the predominance of these serovars in lymph nodes (L54, references 4, 9, 11). We also mentioned that these findings suggest these serovars may be better adapted to lymph nodes (L54-55). Apparently, the text was not clear enough. In any case, the whole introduction was modified following previous reviewers’ comments. We hope the new version is specific enough in this regard.

***RC30****. Lines 57-60. I think you need to do a more thorough search on Salmonella population dynamics using whole genome sequencing. There are several studies that have done this. This is not the first study to compare Salmonella from different sources using WGS.*

**AR31.** We did not claim to be the first study on this topic. We did not refer either to general *Salmonella* population dynamics using WGS. This research deals with *Salmonella* populations circulating in lymph nodes from apparently healthy cattle and its relevance for ground beef contamination (as stated in L50-51).

In this specific context, comparative genomics of the virulence gene repertoire of strains from lymph nodes and ground beef have been poorly addressed (as mentioned in L56-57). We also mentioned that few studies (we did not state that no studies) have conducted both phylogenetic analysis and evolutionary dynamics of these specific *Salmonella* populations.

The term “evolutionary dynamics” should be understood as the reconstruction of character state evolution, which is the isolation source, as explained in **AR16** above). In this regard, we are sure this is one of the few studies addressing these topics altogether (genetic relatedness, character state evolution analysis, and virulence profile) in isolates collected from epidemiologically related samples of lymph nodes and ground beef. The authors agreed, however, to revise the cited papers and the updated manuscript now cites several additional publications, including doi: 10.1016/j.foodcont.2014.09.011 and doi: 10.1128/aem.00033-23.

***RC32****. Methods. Lines 67-76. Need to provide a more detailed description of the sampling population. You mention these samples were from a carcass retailer in Mexico City but need to provide a description of where these carcasses would have originated. How large of an area does this retailer obtain cattle from.*

**AR32.** This remark agrees with ***RC4*** made by reviewer #1. As mentioned in **AR4** above, we agree these details are absolutely required. We did not describe them in full to avoid repeating ourselves with the description provided in previous papers of the same project that we have published (i. e. citation #9 in L74). However, we agreed to describe all the methods here as well, for the sake of clarity. Please, refer to the revised manuscript.

***RC33****. Lines 67-76*

*Also for the samples, you mention the total number of samples collected in that year and the number of isolates per serovar that were chosen for this study. Was this all the Salmonella isolated from the samples or only a subset. If a subset, how was that subset chosen.*

**AR33.** We analyzed all the isolates obtained from the 1,545 samples collected in our previous survey, not a subset. Following previous comments, we adjusted the text to provide all the important details on the origin of the isolates. Please, refer to the updated manuscript.

***RC34****. WGS – Lines 79-90. For DNA extraction, need to specify whether you followed manufacturer protocols. Need to include manufacturer and location for all products and equipment. For instance no manufacturer provided for Qubit or Nextera XT kit. Also need to specify if followed manufacturer protocol for Nextera XT library prep kit. What NextSeq sequencing kit was used?*

**AR34.** This section was modified to ensure all these details are provided.

***RC35****. Line 93 - what was the criteria? The Q score mentioned below? Perhaps place it in this sentence instead.*

**AR35.** Accepted. We adjusted the text to make sure the quality criteria used for trimming, as well as the whole trimming strategy are fully described. Please, refer to the revised manuscript.

***RC36****. Line 100- so sequencing data from these isolates has already been reported? How is this study different than the previous study. Why not state earlier that these isolates had been previously sequenced and cite the paper. I assume this paper goes further into the characterization of those previously reported isolates?*

**AR36.** Yes, we reported the sequencing data from these isolates in a previous paper (reference 17). However, that paper focused on antimicrobial resistance and included all the *Salmonella* genomes from Mexico (n=2,400) that were uploaded at NCBI at that moment. The topic is not related to that of this manuscript, which is the reason why we did not find a right place to cite it in a previous section. However, considering previous comments, both the introduction and the methods sections now includes a description of the origin of these isolates and cites the two papers that were published before.

***RC37****. Line 107 – Why would you use a Salmonella Typhi reference to compare to non-typhoidal Salmonella isolates? If you have several different serotypes it would be good to include references of each of the serotypes since your phylogenetic tree is going to branch by serotype first.*

**AR37.** This comment was also made by the other reviewer (***RC14***). The program we used (CSI Phylogeny) can be run with raw reads or assembled genomes. We used assembled genomes to avoid working with the bulky raw reads (see a more detailed explanation in **AR14** above). Hence, it does not force query genomes to match the sequence of the reference, and thus, no data is discarded during the analysis. Instead, the program calculates the SNP distance between each query genome and the reference genome. This allows calculating the SNP distance between any two genomes (all possible pairs) and building the SNP matrix, which is one of the output files. In fact, we could have selected any genome as a reference, but we chose that of *Salmonella* Typhi to use it as an outgroup in the phylogenetic analysis. This genome was later removed since we mapped the virulence profile onto the phylogenetic tree, and there was no reason to report that of the reference/outgroup Typhi strain. In the updated manuscript, the text was adjusted to ensure these methods are fully described and we hope they are clearly explained. Moreover, the phylogenetic tree now includes the Typhi strain, although no virulence data for this strain is reported in Fig.1.

***RC38****. Line 121 – virulence factor database should be capitalized.*

**AR38.** Thank you for this remark. This mistake was corrected in the revised manuscript.

***RC39****. Results. Did you use WGS to determine serovars. You start discussing serovars in relation to the phylogenetic tree but we have no background on the number of each serovar. You need to provide information on the serovar distribution in the results if you used WGS to determine. If serotyping was completed in the previous study, you should describe the number of each serovar that were included in this study for characterization. Also how did serotypes differ by sample type?*

**AR39.** Yes, the serovar was predicted *in silico*, as reported in our previous paper (reference #9 cited on L74) using the raw reads in the SeqSero program. However, we did not start the results section by discussing the serovars. Instead, we started describing results of the phylogenetic analysis. In this regard, one of the evident results is that isolates of the same serovar (except two) clustered together. We agree with this reviewer that this comment was raised by the lack of information we provided about the study isolates (we only cited the previous paper). The updated manuscript now provides all the necessary background information on the study isolates to facilitate the interpretation of results.

***RC40****. Line 121 – virulence factor database should be capitalized.*

**AR40.** This comment is the same as **RC38**. It was corrected in the revised manuscript.

***RC41****. For the phylogenetic analyses you mention clustering by serotype but you do not mention sample types. You only mention sample type (LN versus ground beef) in relation to evolutionary dynamics.*

**AR41.** We disagree with this comment. In the statement of L146-148, we mentioned that “we observed clonality (100% bootstrap support) between isolates of the same serovar across clusters, regardless of their isolation source”. However, we corrected the text to be more explicit in this regard, and we also included results of the SNP distance between isolates across isolation sources and collection years. Please, refer to the revised manuscript.

***RC42****. Lines 157-160. I do not understand the x-axis scale for this figure and what this text describes. What is a character state and how do you know if remains unchanged or changes among the branching?*

**AR42.** We are sorry for the bad quality of the figures included in the built PDF. We believe this poor quality led to several reviewers’ comments. Our original figures are of good quality, as well as their *.tif versions obtained with the PACE tool. However, the resolution was lost when the PDF was built during submission. Anyhow, as mentioned before, it is possible to download and visualize the original figures by clicking the blue link on top of each figure’s page. Meantime, here is the answer to this comment.

This analysis is a reconstruction of character state at ancestral nodes. This term “character state” was defined in the methods section (L115-116) of the original manuscript, as well as in the caption of Fig. 2 (L162-166). Though blurred, it was also indicated at the top of the figure legend (on the left: “Character 1: isolation source”). The x-axis scale reports the number of ancestral nodes (12 in total). As it is a reconstruction analysis, it goes from 0 (the tip branches on the extreme right of the tree) to 12 (extreme left node that corresponds to the common ancestor of all study isolates). Moreover, the tree should be read from right to left (from node 1 to 12) to see what the estimated isolation source of the isolates at each ancestral node was.

The Mesquite software paints the tree branches according to the levels of the estimated character state: lymph nodes (gray) and ground beef (black). Though the image was blurred, this was indicated in the figure legend, and it is also explained in the figure caption. The branches closest to the tips are color coded according to the actual isolation source, whereby those going backwards (to ancestral nodes) are color coded according to the reconstructed isolation source of ancestors at each node. In this way, it is possible to see whether the estimated isolation source changes across the ancestral nodes. Once the original figure is visualized, you will be able to see that the reconstructed isolation source of the ancestors of Typhimurium isolates (lowest branches of the upper clade) remains unchanged (ground beef) from node 2 to 8. From that node backwards (8-12), it is estimated to be lymph nodes.

A similar comment was raised by the other reviewer. Hence, we adjusted the methods section according to both comments to ensure the methods provide all the necessary details to understand the analysis. We hope these corrections in the text and the consultation of the original figure files would solve this issue.

***RC43****. No statistical analysis was completed. You mention trends between genes and serotypes but did not provide any stats as to whether these associations are significant.*

**AR43.** We do not understand this comment as we did not use the term “trend” in the manuscript. We guess that this reviewer is perhaps referring to the results we reported under the “Virulence and pathogenicity islands” subheading (L169-179, L180-183). However, in this section, we are only describing the results (presence/absence) of certain virulence genes or SPIs in the genomes of isolates of specific serovars. We did not claim there was a trend of any nature here. This is just what we found, and we provided a summarized report of what the virulence heatmap and the BLAST atlas are showing.

For instance, in L182-183, we mentioned that the SPI profile was similar across serovars. This statement is consistent with the results depicted in the BLAST atlas, which shows that nearly all isolates (as mentioned in L183) had the same SPI profile, except for the singletons of serovars Give and Muenster. These results do not suggest the need to conduct any statistical analysis to test if there was an association between specific SPI profiles and serovars. Especially, considering that only the Give isolate lacked most of the SPI-6 sequence, while that of serovar Muenster lacked just a fraction of it.

Another guess we can make is that the reviewer is referring to the statement of L261 (discussion on virulence and pathogenicity islands): “*Most variation occurred between serovars*”. However, this statement is just recalling information from the results section for the sake of discussion, which is then elaborated from L262-269. The statistical support of these results is given by the e-value threshold we used to consider a positive hit for virulence genes (10^-30^, as declared in L124 of the methods section), and for the SPIs sequences in the BLAST atlas analysis: 10^-4^. This last e-value was not actually reported in the original manuscript as we mentioned to have used default values for the BLAST atlas analysis (L133). However, this is now fully explained in the revised manuscript.

***RC44****. Discussion. How can you determine that the LNs are the most likely ancestors of isolates circulating in ground beef when you did not include other sample types in your analysis (fecal, hide, etc.)*

**AR44.** As mentioned in L221, this interpretation came from the results of the character state reconstruction analysis. Moreover, the first paragraph of the discussion starts with the phrase “In this study” (L219). Therefore, this claim should be interpreted in the context of our study. We did not mean to generalize. Our character state reconstruction analysis only included isolates collected from lymph nodes and ground beef, which were the samples we could collect simultaneously at retail. In L232-233, we recognized the limitations associated with not having access to other sample types (i. e. feces), which did not allow us to assess their epidemiological role in the contamination of our ground beef samples. However, we hypothesized and discussed, based on previous research, that isolates circulating in both lymph nodes and ground beef may be of fecal origin (L226-247). We also recognized the need for further research in this area (L245-247). Anyhow, the whole discussion section was modified to gain in clarity and ensure claims are supported by our results.

***RC45****. Much of your discussion is speculative and although you provide references, you do not show how your data and study supports or refutes these statements. This is specifically with regards to the first few paragraphs about LNs and ground beef.*

**AR45.** The first paragraph is just recalling the results from phylogenetic and character state reconstruction analyses. We used these results as the starting point for the discussion. These analyses were performed with isolates from LNs and ground beef from the same carcasses. Though isolates from both sources were collected and analyzed separately, many of them were still clonal, and isolates from both sources were estimated to have ancestors originated from LNs. Since we could not analyze any other sample type at retail, the only thing we could do was hypothesized that perhaps this close relationship is due to their common origin (i. e. feces, which is a well-known source of *Salmonella* according to mainstream science). We do not have results to support or refute these statements. That is why we recognized (L242) that “it is reasonable to speculate…” and that further research in this area is required (L247, recognizing this as a limitation of our study). However, we appreciate this comment and the previous ones, which helped us improve the discussion (see major changes in the revised manuscript).

***RC46****. Lines 260-269. – You should also discuss host-adapted versus host-general serotypes. There is a body of literature out there that shows certain serotypes are consistently found to be carried asymptomatically in cattle and there are other serovars associated with Salmonellosis in cattle. Salmonellosis is also more common in calves than adult cattle and these would be adult cattle.*

**AR46.** We partially agree with this comment. None of the serovars we detected are host-adapted. They are all generalists and have been isolated from many other animal species, vegetables and surface waters in Mexico. Our study intentionally looked for strains carried by asymptomatic animals sent for slaughter since they are more relevant for the “silent” dissemination of foodborne pathogens. Moreover, the reviewer should consider that serotyping was not an objective of this research. Serotyping results were address in our previous paper (reference #9). However, we agreed to enrich the discussion about the serovars, but just considering what is relevant within the scope of our study. Please, refer to the revised manuscript.

***RC47****. How do the virulence profiles by serotype relate to serotypes commonly associated with salmonellosis? For instance the top reported serovars associated with salmonellosis in humans. Do you have data for these serovars and did they tend to have more virulence genes?*

**AR47.** We built the virulence profile based on the reference sequence of major *Salmonella* virulence factors and 12 SPIs. These are the data supporting the capability of the pathogen to infect mammals, as we mentioned in L281. In this regard, our results showed that practically all isolates carried a conserved virulence repertoire, which is consistent with the implication of all these serovars in human salmonellosis in Mexico. The last part (the underlined text) was not included in the discussion as it repeats itself with our previous paper (reference #9), but we enriched this analysis, as mentioned in our response to the previous comment (**AR46**).

***RC48****. Lines 277-283 – Have other studies found similar SPI profiles among these serotypes. Are SPIs very serotype specific or does this differ from study to study?*

**AR48.** We agree the discussion of this topic should be enriched. Please, refer to the revised manuscript.

***RC49****. While I think it is important that the Salmonella in this study are genomically similar to strains involved in cases of salmonellosis, I believe it is also important to put this into context. These strains are from different geographical locations and at different time points and this needs to be stated along with other reasons that these strains may be similar.*

**AR49.** We agree and appreciate this comment. We mentioned that these strains only pose a risk to public health (we did not claim they were implicated in cases of human salmonellosis). However, this is a good observation. Hence, the discussion was adjusted to make this clear.

***RC50****. Lines 294-303 – the language is a bit strong here regarding what you can extrapolate with your data. I think your data supports that Salmonella from LNs are closely related to Salmonella from ground beef and LNs may be a reservoir for Salmonella in ground beef. Your data does show that Salmonella are found in healthy animals at slaughter which can contribute to Salmonella entering the food chain, though others have shown that many serotypes are carried in cattle without causing disease and this is not something novel.*

**AR50.** We carefully revised this fragment to avoid claims that are not supported by our results. We hope that, after the adjustments made, and the better description of the isolates we now provide in the methods, the reviewer will discard this observation.

***RC51****. Need to acknowledge limitations of the study.*

**AR51.** We carefully revised the text to make sure the limitations were properly acknowledged. Please, refer to the revised manuscript.

***RC52****. Figure 1 is very blurry. Tough to read if ground beef or LN and also the genes along the top. Should color text of beef/LN different from amino acid identity color. Why did you choose a heatmap over using a cutpoint and presence/absence? Looks like all values are 0, 50, or 100.*

**AR52.** This observation relates partially to the poor resolution of the figure. As mentioned before, the PDF building process yields figures of poor quality. To check the good ones, you must download the original file from the blue download link on top of each figure’s page. We changed the color code of beef and LNs, although the isolation source is also indicated on the tip labels. In our experience, using the presence/absence approach may lead to lose information. For instance, in one of our previous papers (doi: 10.1038/s41598-018-28169-4), we looked for *sodCI*, which protects *Salmonella* from the host oxidate burst (is thus associated with intracellular lifestyle). Some isolates did not carry *sodCI* but *sodCII*, which share <60% amino acid sequence identity with *sodCI*. The SodCII protein protects the pathogen from the oxidative damage in the extra-cellular environment. If we had used a cutoff value (e.g., 80% amino acid identity), would have missed the presence of the *sodCII* gene, which was also a relevant finding. Not all values are 0, 50 or 100. Again, the poor resolution of the figure in the built PDF did not help in this regard. In fact, several protein-encoding genes had variations in their amino acid sequence (especially adhesion factors, the SPI-4 *siiE* and *iroN*). Thus, desides changing the color code of the isolation sources, we have included the results used for the heatmap as supplementary information.

***RC53****. Figure 2 is very blurry. Hard to decipher LN versus ground beef. Also would be nice to color code by serovar. What is the scale along the x-axis represent.*

**AR53.** This observation relates to the poor resolution of the figure in the built PDF, which we explained before. The serovars are indicated at tip labels. We did not use a color-code for serovars to avoid moving the focus from the ancestral state reconstruction (which is the isolation source, represented by black and grey color). We believe using so many colors could confuse the readers. Hopefully, this observation will be considered solved after checking the original figure files and its caption.

***RC54****. Figure 4 – I cannot read this figure and I have no idea how to interpret this information.*

**AR54.** This observation relates mostly with the poor resolution of the figure in the built PDF. Its caption provided a description of what it shows though (L211-216), and there was also a brief explanation in the methods section (L134-139). The right side of each figure shows the isolates that formed each SNP cluster (we highlighted in red our study isolates and those involved in clinical cases). The left side of each figure reports the SNP cluster accession number (on the top), the number of isolates that were selected, the SNP distance between them (minimum, average, and maximum), and some metadata.

We also described that these SNP clusters demonstrated the genetic proximity between our study isolates and those involved in human salmonellosis (L199-209) and provided the full list of SNP clusters analyzed in this study as supplementary information (L138-139, S1 Table). Following this comment, we discarded the original figure. Instead, we downloaded (from the NCBI Pathogen Detection website) the phylogenetic tree of one SNP cluster containing our isolates and clinical strains. The tree was visualized in FigTree and exported in SVG format to edit it using IncScape. We hope this observation will be considered solved after checking the updated figure and caption, as well as the improved description of the methods.
