# Supplementary material for: Long-term genomic surveillance reveals the circulation of clinically significant Salmonella in lymph nodes and beef trimmings from slaughter cattle from a Mexican feedlot
Source: PLoS One. 2024 Oct 18;19(10):e0312275. doi: 10.1371/journal.pone.0312275 (PMC11488740; doi:10.1371/journal.pone.0312275)
Supplement: Attachment — Submitted filename: PONE review.docx [file pone.0312275.s007.docx]

PONE-d-24-16621

L20. How was the *Salmonella* “circulating” in the cattle? No data were collected from the cattle – only from carcass tissues.

L21. Based on methodology, “ground beef” was not sampled. Instead 200 g of lean meat from primal cuts that are frequently used for ground beef production were analyzed. Therefore, the title and discussion is misleading and does not represent typical ground beef. This is a major concern and is misleading.

L 27-30. The sentence starting with “Although lymph nodes…” is unlcear.

L 52. USA - consistency

L 59. US – consistency

L 55-56. Delete “Evidence from this” and start sentence with Research.

L 60. What does “usually overrepresented” mean? If these are the most common ones found, then just state that.

L 71. Unclear which study is being referred to by “this study”

L 76. Which populations are referred to by “…of these *Salmonella* populations?”

L 78 – 81 . “In this research” and “During a previous study” are both used. Seems like it would have to be one or the other but not both. Please clarify.

L 83. Ground beef samples were not obtained – based on L 119-120.

L 124-132. Not clear if samples were from same carcasses or not.

L 548 - 554. How can conclusions about “asymptomatic cattle”(L548 and 553) be made when no data were collected from live cattle? This is overstating the findings and drawing conclusions that are not supportable.

L 560-562. Again no data were collected on live cattle, so statements about “transmission dynamics of *Salmonella* among healthy cattle” should not be made.

L 563 – 565. Data presented do not support that *Salmonella* persists for long periods in farm environments. No environmental farm data were collected or analyzed.

Overall:

The authors need to carefully review their discussion. Many of the statements are not related to the data collected or presented and overstate the authors findings. This is a major concern and should be addressed before publication.
